# The prebiotic inulin modulates gut microbiota but does not ameliorate atherosclerosis in hypercholesterolemic APOE*3-Leiden.CETP mice

**DOI:** 10.1038/s41598-018-34970-y

**Published:** 2018-11-08

**Authors:** Lisa R. Hoving, Saeed Katiraei, Amanda Pronk, Marieke Heijink, Kelly K. D. Vonk, Fatiha Amghar-el Bouazzaoui, Rosalie Vermeulen, Lizette Drinkwaard, Martin Giera, Vanessa van Harmelen, Ko Willems van Dijk

**Affiliations:** 1 Department of Human Genetics, Leiden University Medical Center (LUMC), 2300 RC Leiden, The Netherlands; 2 Einthoven Laboratory for Experimental Vascular Medicine, Leiden University Medical Center (LUMC), 2300 RC Leiden, The Netherlands; 3 Center for Proteomics and Metabolomics, Leiden University Medical Center (LUMC), 2300 RC Leiden, The Netherlands; 4 Department of Medicine, division Endocrinology, Leiden University Medical Center (LUMC), 2300 RC Leiden, The Netherlands

## Abstract

Gut microbiota have been implicated in the development of atherosclerosis and cardiovascular disease. Since the prebiotic inulin is thought to beneficially affect gut microbiota, we aimed to determine the effect of inulin supplementation on atherosclerosis development in APOE*3-Leiden.CETP (*E3L.CETP*) mice. Female *E3L.CETP* mice were fed a western-type diet containing 0.1% or 0.5% cholesterol with or without 10% inulin. The effects of inulin were determined on: microbiota composition, cecal short-chain fatty acid (SCFA) levels, plasma lipid levels, atherosclerosis development, hepatic morphology and hepatic inflammation. Inulin with 0.5% dietary cholesterol increased specific bacterial genera and elevated levels of cecal SCFAs, but did not affect plasma cholesterol levels or atherosclerosis development. Surprisingly, inulin resulted in mild hepatic inflammation as shown by increased expression of inflammation markers. However, these effects were not accompanied by increased hepatic macrophage number. Analogously, inulin induced mild steatosis and increased hepatocyte size, but did not affect hepatic triglyceride content. Inulin with 0.1% dietary cholesterol did not affect hepatic morphology, nor hepatic expression of inflammation markers. Overall, inulin did not reduce hypercholesterolemia or atherosclerosis development in *E3L.CETP* mice despite showing clear prebiotic activity, but resulted in manifestations of hepatic inflammation when combined with a high percentage of dietary cholesterol.

## Introduction

Atherosclerosis is a narrowing of arteries due to accumulation of lipids and cells in the intima, leading to cardiovascular diseases (CVD) including heart attack and stroke. CVD are a leading cause of morbidity and mortality in Western Society^1^. Hypercholesterolemia is one of the main underlying risk factors of atherosclerosis development, and is routinely treated by prescription of statins. However, statin treatment lowers total plasma cholesterol levels by approximately 30%^2^ and only prevents 25–45% of all cardiovascular events^3^, indicating the demand for additional therapies. The gut microbiota have been discovered as an important player in the onset of atherosclerosis and CVD^4^. Disturbances in lipid metabolism, the precursor for the development of atherosclerosis and CVD, have also been associated with gut microbiota dysbiosis in both rodents^5^ and humans^6^. A well-known factor that modulates the gut microbiota composition and function are dietary fibers or prebiotics. Inulin is a widely studied prebiotic that has been shown to beneficially modify gut microbiota composition and function^7^. Inulin is fermented by specific bacteria in the gut, leading to outgrowth of these bacteria and short-chain fatty acid (SCFA) production^8^, which is thought to improve colonic and systemic health^9^. Inulin has been shown to exert multiple beneficial effects on the host, including lowering inflammation^10,11^, as well as decreasing hyperlipidemia^12–15^. Whether inulin affects the development of atherosclerosis is currently underexplored. Therefore, we set out to determine the effect of inulin on the development of atherosclerosis in hypercholesterolemic APOE*3-Leiden.CETP (*E3L.CETP*) mice, a model that is characterized by a human-like lipoprotein metabolism and that is susceptible to the development of atherosclerosis. Particularly female *E3L.CETP* mice are highly sensitive to dietary cholesterol and they respond in a human-like manner to lipid-lowering anti-atherogenic therapies^16^. We found that inulin drastically altered gut microbiota composition and function. However, inulin neither decreased hypercholesterolemia nor ameliorated atherosclerosis development in this mouse model. Notably, inulin in combination with a high percentage of dietary cholesterol resulted in manifestations of hepatic inflammation.

## Results

### Inulin modified gut microbiota composition and function

**Figure 1:**
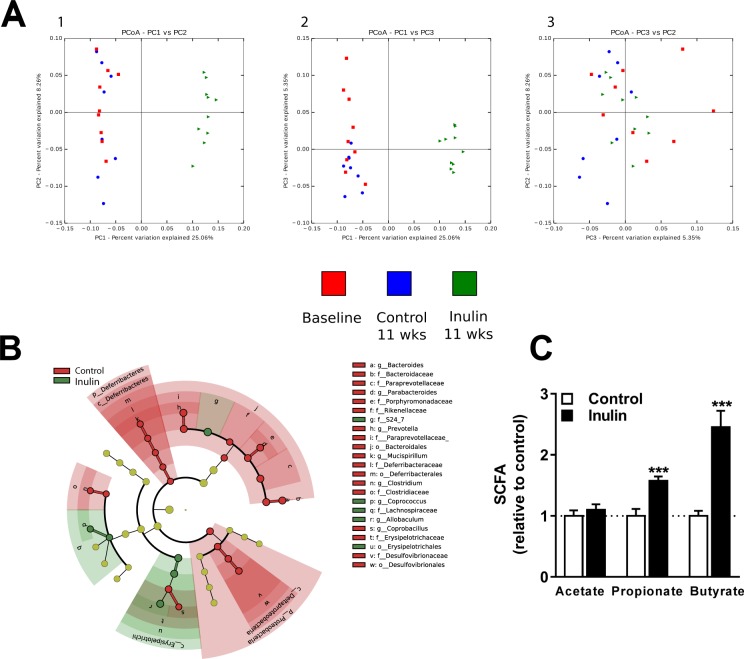
Inulin modified gut microbiota composition and function. The effect of inulin supplementation on microbiota composition and function in mice fed a WTD containing 0.5% cholesterol ± inulin. (**A**) Principal coordinates analysis (PCoA) plot of unweighted UniFrac distances metric of 16S rRNA gene sequences at baseline (depicted in red) and for the subsequent control group (depicted in blue) and inulin group (depicted in green) after 11 weeks (wks) of treatment. To evaluate similarities between bacterial communities, graphs 1, 2, and 3 were generated using OTU’s based on the unweighted UniFrac distance metrics PC1 and PC2, PC1 and PC3, and PC3 and PC2, respectively, and are shown on multiple two-dimensional arrays. (**B**) The cladogram reports the taxa (highlighted by small circles and by shading) showing relative abundance values with a maximum depth to genus level (according to LEfSe). Red or green circles/shading represents taxa that are significantly higher in relative abundance, while yellow indicates taxa which did not differ in relative abundance between the two groups. The taxonomic level is indicated by letter preceding the underscore: o, order; f, family; g, genus. The central point in the cladogram represents the taxonomic domain ‘bacteria’ and each ring outward represents the next lower taxonomic level (phylum to genus). The darker the shading of the red or green colours, the higher the abundance (n = 8–10 mice per group). (**C**) Finally, the cecal SCFAs acetate, propionate, and butyrate were determined (n = 10 mice per group). Values are presented as means ± SEM. *P < 0.05, **P < 0.01, ***P < 0.001 vs. control.

To determine the effects of inulin on gut microbiota in mice fed a WTD with 0.5% cholesterol, 16S rRNA gene sequencing was used to assess the effect of inulin on gut microbiota composition and relative abundance of specific gut microbial taxa. Cluster analysis based on unweighted UniFrac distances revealed a clear difference between the control group and the inulin group after 11 weeks of intervention, while the control group did not change after 11 weeks compared to the baseline measurement (Fig. 1A).

Further analysis of the gut microbiota revealed clear differences in the composition of the microbial community between the control group and the inulin group. Figure 1B schematically depicts LEfSe’s results included in a cladogram showing the significant differences on each taxonomic level with a maximum depth to genus level. Table 1 shows the relative abundances (%) of genera in the control and the inulin group and the percentage difference between the two groups based on LEfSe and ANCOM analyses. Based on LEfSe analysis, inulin significantly increased the relative abundance of the genera *Coprococcus* (+409%), and *Allobaculum* (+833%), whereas the genera *Bacteroides* (−59%), *Parabacteroides* (−60%), *Prevotella* (−88%), *Micispirillum* (−100%), *Clostridium* (−100%), and *Coprobacillus* (−100%) were reduced compared to control mice (Table 1). Based on ANCOM analysis, inulin increased the relative abundance of the genera *Coprococcus* (+409%), *Ruminococcus* (+52%), *Allobaculum* (+833%), and *Sutterella* (+12%), whereas the genera *Mucispirillum* (−100%) and *Coprobacillus* (−100%) were decreased compared to control mice (Table 1). The overlapping genera that significantly increased after inulin supplementation compared to control mice according to both analyses were *Allobaculum* and *Coprococcus*.

**Table 1:** Relative abundance and the percentage difference of the inulin group (n = 10) and the control group (n = 8) for each genera at 11 weeks of intervention. Data are presented as mean ± standard error of the mean (SEM).

Phylum	Class	Order	Family	Genus	Control	Inulin	Inulin/Control
					Abundance (%)	Abundance (%)	% difference
					Mean ± SEM	Mean ± SEM	Mean ± SEM
Bacteroidetes							
	Bacteroidia						
		Bacteroidales					
			Bacteroidaceae				
				Bacteroides	15.53 ± 1.87	6.33 ± 1.00	−59%*
			Porphyromonadaceae				
				Parabacteroides	8.88 ± 1.60	3.52 ± 0.71	−60%*
			Paraprevotellaceae				
				Prevotella	17.2 ± 6.29	2.1 ± 2.03	−88%
			Rikenellaceae				
				*Unidentified*	*3.28* ± *0.90*	*0.06* ± *0.02*	*−98%*
			S24-7				
				*Unidentified*	*5.54* ± *0.90*	*44.88* ± *2.76*	*710%*
			*Unidentified*				
				*Unidentified*	*3.21* ± *0.68*	*0.03* ± *0.02*	*−99%*
Deferribacteres							
	Deferribacteres						
		Deferribacterales					
			Deferribacteraceae				
				Mucispirillum	1.83 ± 0.75	0 ± 0	−100%
Firmicutes							
	Bacilli						
		Lactobacillales					
			Lactobacillaceae				
				Lactobacillus	5.69 ± 2.06	3.27 ± 0.76	−43%
	Clostridia						
		Clostridiales					
			Clostridiaceae				
				Clostridium	1.52 ± 0.70	0 ± 0	−100%*
			Lachnospiraceae				
				Coprococcus	0.38 ± 0.12	1.91 ± 0.45	403%*^$^
				Ruminococcus	1.08 ± 0.36	1.64 ± 0.44	52%^$^
			Ruminococcaceae				
				Oscillospira	2.08 ± 0.60	0.96 ± 0.21	−54%
				*Unidentified*	*1.48* ± *0.85*	*0.48* ± *0.11*	*−68%*
			*Unidentified*				
				*Unidentified*	*14.65* ± *3.37*	*12.85* ± *2.46*	*−12%*
	Erysipelotrichi						
		Erysipelotrichales					
			Erysipelotrichaceae				
				Allobaculum	1.88 ± 0.55	17.50 ± 2.30	831%*^$^
				Coprobacillus	1.17 ± 0.44	0 ± 0	−100%*^$^
				*Unidentified*	*5.86* ± *1.93*	*0* ± *0*	*−*100*%*
Proteobacteria							
	Betaproteobacteria						
		Burkholderiales					
			Alcaligenaceae				
				Sutterella	2.34 ± 0.80	2.63 ± 0.55	12%^$^
	Deltaproteobacteria						
		Desulfovibrionale					
			Desulfovibrionaceae				
				*Unidentified*	*4.78* ± *1.54*	*1.52* ± *0.71*	*−68%*
	Gammaproteobacteri						
		Enterobacteriales					
			Enterobacteriaceae				
				*Unidentified*	*1.61* ± *0.75*	*0.32* ± *0.17*	*−80%*

*P < 0.05 according to LEfSe analysis; ^$^P < 0.05 according to ANCOM analysis.

Subsequently, we determined the effects of inulin on SCFA levels in the cecum. Inulin increased cecal levels of propionate (+57% vs. control; P = 0.0005) and butyrate (+146% vs. control; P = 0.0002), but not of acetate (Fig. 1C). These data indicate that dietary inulin supplementation modulated both microbial composition and function in *E3L.CETP* mice.

### Inulin neither decreased hyperlipidemia nor ameliorated atherosclerosis development

**Figure 2:**
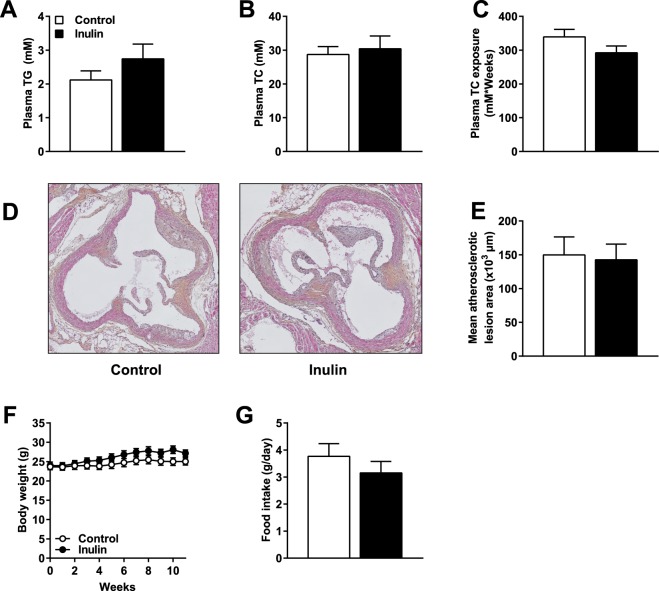
Inulin neither decreased hyperlipidemia nor ameliorated atherosclerosis development. Mice were fed a WTD containing 0.5% cholesterol ± inulin for 11 weeks. (**A**) Plasma TG levels, (**B**) plasma TC levels, and (**C**) cumulative TC exposure were determined in 4 hour fasted mice. (**D**) Representative cross-sections of the valve area of the aortic root of the heart stained with HPS are shown, and (**E**) the mean atherosclerotic lesion area from the four consecutive cross-sections. Finally, (**F**) body weight, and (**G**) food intake were determined. Values are presented as means ± SEM (n = 10 mice per group). P < 0.05 was considered as statistically significant.

Given the potential beneficial effects of inulin on plasma lipid parameters, we determined the effect of inulin on plasma triglycerides (TG) and total cholesterol (TC) levels in *E3L.CETP* mice fed a Western-type diet (WTD) containing 0.5% cholesterol ± inulin. Surprisingly, inulin did not affect plasma TG levels (Fig. 2A), plasma TC levels (Fig. 2B), or TC exposure (Fig. 2C). In order to determine whether inulin affected atherosclerosis development, we measured atherosclerosis progression in the aortic root of the heart after 11 weeks of inulin supplementation. Representative images (Fig. 2D) illustrate that inulin did not affect mean atherosclerotic lesion area throughout the aortic root (Fig. 2E). Finally, there was no effect of inulin on body weight (Fig. 2F) or food intake (Fig. 2G) in these mice. These data show that inulin did not beneficially lower plasma lipid levels and did not affect atherosclerosis development in hypercholesterolemic *E3L.CETP* mice.

### Inulin adversely affected the liver by manifestations of hepatic inflammation

**Figure 3:**
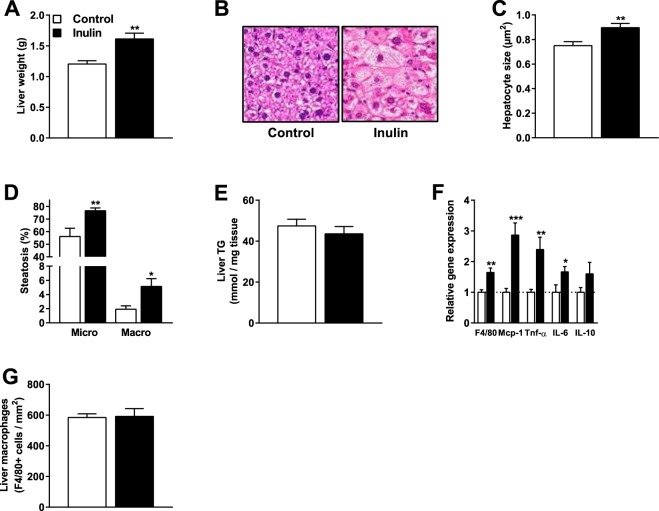
Inulin adversely affected the liver by manifestations of hepatic inflammation Mice were fed a WTD containing 0.5% cholesterol ± inulin for 11 weeks. (**A**) Liver weight, (**B**) liver morphology, (**C**) hepatocyte size as a marker for hepatic hypertrophy, (**D**) microvesicular and macrovesicular steatosis, (**E**) liver TG content, (**F**) qRT-PCR analysis of *F4/80*, *Mcp-1*, *Tnf-α*, *IL-6*, *IL-10*, and (**G**) immunohistochemical analysis of hepatic *F4/80* were determined. Values are presented as means ± SEM (n = 10 mice per group). *P < 0.05, **P < 0.01, ***P < 0.001 vs. control.

Although inulin did not affect hyperlipidemia and atherosclerosis, we found that inulin increased liver weight in mice fed a WTD containing 0.5% cholesterol (+34% vs. control; P = 0.003; Fig. 3A). We observed notable hepatic morphological derangements in the inulin group (Fig. 3B). Inulin significantly increased hepatic hypertrophy as indicated by increased hepatocyte size (+20% vs. control; P = 0.008; Fig. 3C). Inulin increased microvesicular steatosis (+37% vs. control; P = 0.006) and macrovesicular steatosis (+167% vs. control; P = 0.03)(Fig. 3D), but did not affect hepatic TG content (Fig. 3E). Hepatic gene expression of *F4/8*0 (+65% vs. control; P = 0.002), *Mcp-1* (+186% vs. control; P = 0.0009), *Tnf-α* (+139% vs. control; P = 0.008), and *IL-6* (+67% vs. control; P = 0.04) were higher in the inulin group without any effect on *IL-1*0 expression, indicating a pro-inflammatory phenotype in the mice fed inulin (Fig. 3F). However, inulin did not lead to changes in the macrophage marker *F4/80* as quantified by immunohistochemical staining (Fig. 3G). These data indicate that inulin in combination with 0.5% dietary cholesterol adversely affected liver morphology and resulted in manifestations of hepatic inflammation in hypercholesterolemic *E3L.CETP* mice.

### Inulin in combination with 0.1% dietary cholesterol increased SCFAs in cecum content, but did not affect hyperlipidemia or hepatic inflammation

**Figure 4:**
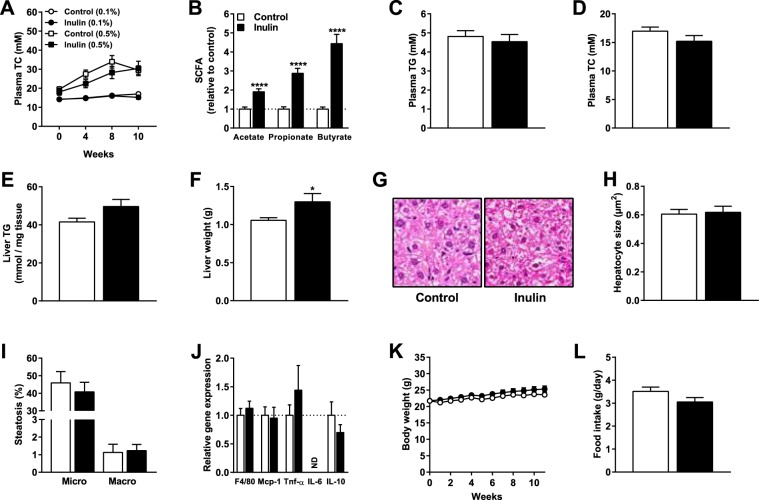
Inulin in combination with 0.1% dietary cholesterol increased SCFAs in cecum content, but did not affect hyperlipidemia or hepatic inflammation. Mice were fed a WTD containing 0.1% cholesterol ± inulin for 11 weeks (n = 17 mice per group). (**A**) Plasma TC levels for control and inulin fed mice on both 0.5% dietary cholesterol (n = 10 mice per group) and 0.1% dietary cholesterol for 0, 4, 8, and 10 weeks of dietary intervention. (**B**) The cecal SCFAs acetate, propionate, and butyrate, (**C**) plasma TG levels, (**D**) plasma TC levels, (**E**) liver TG content, (**F**) liver weight, (**G**) liver morphology, (**H**) hepatocyte size, (**I**) microvesicular and macrovesicular steatosis, (**J**) qRT-PCR analysis of *F4/80*, *Mcp-1*, *Tnf-α*, *IL-6*, and *IL-10*, (**K**) body weight, and (**L**) food intake were determined. Values are presented as means ± SEM. *P < 0.05, **P < 0.01, ***P < 0.001, ***P < 0.0001 vs. control. ND = non-detectable.

We subsequently determined whether inulin in combination with a more moderate percentage of dietary cholesterol (0.1%) would lead to different outcomes. Using this lower percentage of dietary cholesterol, hypercholesterolemia is induced to a lower extent compared with 0.5% dietary cholesterol. Indeed, 0.5% dietary cholesterol resulted in final plasma TC levels of 27.6 ± 1.5 mM, while 0.1% dietary cholesterol resulted in plasma TC levels of 15.6 ± 0.3 mM (Fig. 4A). Similar to mice fed 0.5% cholesterol, inulin in combination with 0.1% dietary cholesterol significantly elevated levels of propionate (+188% vs. control; P < 0.0001) and butyrate (+344% vs. control; P < 0.0001) in cecum content (Fig. 4B). However, 0.1% dietary cholesterol with inulin also increased levels of acetate in cecum content (+90% vs. control; P < 0.0001; Fig. 4B). Inulin in combination with 0.1% cholesterol did not affect plasma TG levels (Fig. 4C), plasma TC levels (Fig. 4D), or liver TG content (Fig. 4E), but increased liver weight (Fig. 4F) which was similar to mice fed 0.5% cholesterol. However, in contrast to mice fed inulin with 0.5% cholesterol, inulin combined with 0.1% cholesterol did not affect hepatic morphology (Fig. 4G), hepatocyte size (Fig. 4H), microvesicular or macrovesicular steatosis (Fig. 4I), or hepatic gene expression of inflammatory markers (Fig. 4J). Finally, there was neither an effect of inulin on body weight (Fig. 4K) nor on food intake (Fig. 4L) in these mice. These results show that inulin in combination with 0.1% cholesterol also modulated gut microbiota function, did also not affect hypercholesterolemia, but in contrast to inulin with 0.5% cholesterol did not affect liver inflammation.

## Discussion

Our data show that dietary inulin did not reduce atherosclerosis in *E3L.CETP* mice fed a WTD with 0.5% cholesterol. This may not have been surprising, since plasma lipid levels were not decreased by inulin supplementation. In contrast, inulin in combination with 0.5% cholesterol was associated with manifestations of hepatic inflammation. To further investigate the interaction between dietary cholesterol and inulin on plasma lipid levels and inflammation, we analysed the effect of inulin in combination with 0.1% cholesterol. In this experiment, we found no clear signs of hepatic inflammation but again no effects of inulin on plasma lipid levels. Thus, inulin appears not to have a cholesterol lowering or anti-atherogenic effect in hypercholesterolemic *E3L.CETP* mice.

Rault-Nania *et al*.^17^, found a 35% reduction in atherosclerotic plaque formation in hypercholesterolemic APOE-deficient mice. APOE-deficient mice are characterized by severe hypercholesterolemia due to a virtually completely blocked LDL-receptor mediated lipoprotein remnant clearance^18^. In contrast, *E3L.CETP* mice express a dominant variant of human APOE, which results in a moderately disrupted LDL-receptor mediated lipoprotein remnant clearance^19^. *E3L.CETP* mice are highly responsive to diet-induced hyperlipidemia and atherosclerosis development and have been widely used as preclinical model to study the effects and underlying mechanisms of various human drugs^16^. Furthermore, the APOE-deficient mice in the study of Rault-Nania *et al*. were fed a semi-purified diet based on sucrose, whereas the *E3L.CETP* mice in our study were fed a WTD. Both mouse models are based on C57BL/6 genetic background. However, whether the differential effects of inulin on atherosclerosis development in APOE-deficient mice versus *E3L.CETP* mice are mouse model-specific and/or a result of differences in dietary composition and/or housing conditions remain to be determined.

Nevertheless, inulin led to elevations of the SCFAs propionate and butyrate on both 0.1% and 0.5% cholesterol diets in *E3L.CETP* mice. Both propionate and butyrate have been suggested to have beneficial effects on cholesterol metabolism. Propionate is absorbed in the colon and transported via the portal vein to the liver where it can inhibit cholesterol synthesis^20^. Butyrate is mainly used as energy substrate for colonocytes, and studies in rats have suggested that ingestion of butyrate may lead to reduced hepatic cholesterol synthesis^21^. Furthermore, Wang *et al*.^22^, recently have shown that butyrate improves energy metabolism by reducing energy intake and enhancing fat oxidation by activating brown adipose tissue in *E3L.CETP* mice. Despite elevations of both propionate and butyrate in our study, no lipid lowering effects of inulin were observed. However, we cannot exclude that prior administration of an inulin containing diet may affect the subsequent response to a cholesterol WTD. This further implies that the effects of administration of inulin prior to cholesterol WTD feeding on SCFAs and atherosclerosis development remains to be further investigated.

Interestingly, inulin in combination with 0.5% dietary cholesterol led to manifestations of hepatic inflammation. This effect seemed to be dependent on the amount of cholesterol in the diet as inulin did not adversely affect the liver when combined with 0.1% cholesterol. We found similar detrimental effects of inulin and cholesterol in an inflammation-driven cuff-induced atherosclerosis model, were inulin in combination with 1% dietary cholesterol even resulted in increased plasma cholesterol levels and aggravated atherosclerosis development^23^. We are not the first ones to show adverse effects of dietary fiber on liver inflammation mediated via gut microbiota interactions. In a previous study by Janssen *et al*.^24^, specific modulation of the gut microbiota, by feeding mice the prebiotic indigestible carbohydrate guar gum, promoted liver inflammation and led to worsening of non-alcoholic fatty liver disease (NAFLD) in mice. In this study, the effects of guar gum on liver inflammation could be linked to altered circulating and hepatic levels of bile acids. Whether inulin in our study adversely affected the liver via changes in bile acid metabolism remains to be investigated.

Inulin has been shown to induce inflammation in previous studies, although in these studies inulin mainly exacerbated pre-existing inflammation. For instance Miles *et al*.^25^, reported that diets enriched with inulin further exacerbated the severity of dextran sulfate sodium (DSS)-induced colitis in mice. It is possible that in our study the high-cholesterol diet induced borderline liver inflammation which was exacerbated by inulin.

Inulin is well established to exert bifidogenic effects and the increased abundance of *Bifidobacteria* in the gut microbiota presumably explains the beneficial effects of inulin on health^26^. Notably, inulin in combination with 0.5% dietary cholesterol led to major shifts in microbial composition but there was no significant increase in the relative abundance of *Bifidobacteria*. Instead, according to both LEfSe and ANCOM analysis, inulin stimulated the relative abundance of the genera *Allobacullum* and *Coprococcus*. Catry *et al*.^27^ have previously reported that dietary intervention with inulin-type fructans (ITF) improved endothelial dysfunction in which they found increased abundance of *Allobaculum* in APOE^−/−^ mice. Furthermore, Lee *et al*.^28^, have reported that feeding rats a diet rich in cholesterol specifically increased the abundance of *Allobaculum* and *Coprococcus* compared to a control AIN76A diet. Whether the relative increase in *Allobaculum* in these and our studies was due to inulin supplementation, hypercholesterolemia, or both is not clear. In the study of Lee *et al*.^28^, it was found that *Allobaculum* exhibited a negative correlation with anti-inflammatory genes in the gut, indicating that *Allobaculum* plays a role in inflammation. We found a relative decrease in the abundance of *Bacteroides*, *Parabacteroides*, *Prevotella*, *Mucispirillum*, *Clostridium*, and *Coprobacillus* after inulin supplementation. Whether the absence of *Bifidobacteria*, relative increase of *Allobaculum* and *Coprococcus*, and/or the decrease in relative abundance of certain other genera played a role in the underlying mechanism of the manifestations of liver inflammation after inulin supplementation on a high cholesterol diet, remains to be investigated.

In conclusion, in this study inulin did neither affect hypercholesterolemia nor atherosclerosis development in hypercholesterolemic *E3L.CETP* mice, but rather resulted in manifestations of hepatic inflammation when combined with high percentages of dietary cholesterol. Although inulin is widely acknowledged as a prebiotic with favorable effects on lipid metabolism and CVD, inulin clearly not always exerts beneficial effects. It will be important for future research to decipher potential adverse pathways and mechanisms that are induced by the interaction of inulin with high dietary cholesterol and the gut microbiota. Although the gut microbiota of mice differ significantly from humans, *E3L.CETP* mice respond similarly as patients to a variety of anti-atherosclerotic interventions^23^. Therefore, we interpret our data to indicate caution with the application of inulin to reduce lipid levels in humans.

## Methods

### Mice and diet

**Figure 5:**
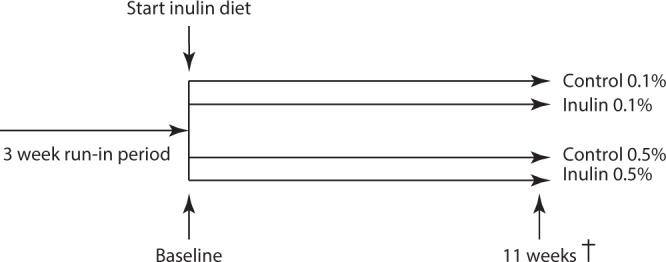
Experimental study protocol. Experiments were performed independently.

In two experiments, female *E3L.CETP* mice were fed a WTD containing 0.1% or 0.5% cholesterol (Diet T 0.1 (Diet T 4022.16) or Diet T 0.5 (Diet T 4022.17); AB Diets, The Netherlands) (Table 2). This WTD diet was supplemented with or without 10% inulin (FrutaFit HD, Sensus, The Netherlands) for a total period of 11 weeks in which 10% of cellulose was replaced with 10% inulin. At baseline, after a run-in of 3 weeks with WTDs, randomization of the mice was performed based on plasma TC levels, plasma TG levels, and body weight. During the intervention period, body weight and food intake were measured weekly. After 11 weeks, non-fasted mice were sacrificed using CO_2_ inhalation. Orbital bleeding was performed to collect blood and mice were subsequently perfused with ice-cold PBS through the heart. Cecum content, heart, and liver were collected for further analysis. Mice were housed under temperature- and humidity-controlled conditions with a 12:12 h light-dark cycle and free access to food and water. A schematic of the experimental study protocol is provided in Fig. 5. Mouse experiments were performed in compliance with Dutch government guidelines and the Directive 2010/63/EU of the European Parliament and had received approval from the University Ethical Review Board (Leiden University Medical Center, The Netherlands).

**Table 2:** Diet composition (% of total weight).

Dietary substitute (%)	Control/Inulin 0.1%	Control/Inulin 0.5%
Inulin	0/10	0/10
Cholesterol	0.1	0.5
Magnesium oxide	0.2	0.2
Methionine	0.2	0.2
Standard trace elements premix	0.25	0.25
Standard vitamin premix	0.25	0.25
Salt	0.3	0.3
Magnesium sulphate heptahydate	0.4	0.4
Potassium hydrogen phosphate	0.7	0.7
Potassium chloride	0.7	0.7
Calcium carbonate	1	1
Corn oil	1	1
Calcium hydrogen phosphate	1.3	1.3
Choline chloride	2	2
Corn starch	10	10
Cacoa butter	15	15
Cellulose	16.1/6.1	16/6
Sour casein	20	20
Sugar/sucrose	30.5	30.2
*Total*	*100*	*100*

### 16S rRNA gene sequencing and profiling

16S rRNA sequencing in cecum samples of *E3L.CETP* mice fed with a WTD (0.5% cholesterol) ± inulin was performed as described previously^29^. Relative bacterial abundance was determined. For statistical significance, biological relevance and visualisation linear discriminant analysis (LDA) effect size (LEfSe) method (https://bitbucket.org/biobakery/biobakery/wiki/lefse)^30^ and ANCOM analysis were used as described previously (https://www.niehs.nih.gov/research/resources/software/biostatistics/ancom/index.cfm)^31^. Prior to LEfse analysis low abundant taxa were filtered out, applying a two-step filtering. First, taxa that were present in less than half of the group size were filtered out. In a second step, very rare taxa that were abundant less than 0.5% of total group taxa were filtered out. At baseline and after 11 weeks (wks) of intervention, jack-knifed β-diversity of unweighted UniFrac distances, with 10 jack-knifed replicates was measured at rarefaction depth of 22000 reads per sample, based on the unfiltered OTU table.

### Cecal short-chain fatty acid analysis

Cecum SCFA content was analysed using gas chromatography mass spectrometry (GC-MS) as described previously^32^.

### Plasma parameters

Plasma TG and TC were measured in 4 hour fasted mice as described previously^29^. Plasma cholesterol exposure was calculated as the cumulative exposure over the number of weeks where mice were fed a WTD ± inulin.

### Atherosclerosis quantification

Atherosclerosis quantification using histological staining with hematoxylin-phloxyin-saffron (HPS) was performed in hearts of *E3L.CETP* mice fed a WTD (0.5% cholesterol) ± inulin as described previously^29^. Image J Software (NIH, USA) was used for the quantification of atherosclerotic lesion areas.

### Liver (immuno)histochemistry

Livers were removed, fixed in 4% paraformaldehyde, dehydrated in 70% ethanol, embedded in paraffin and sectioned (5 µm). Paraffin-embedded liver sections were stained with haematoxylin and eosin (H&E) using standard protocols. From H&E-stained sections, hepatocyte size (hepatic hypertrophy) was quantified as the average number of hepatocytes per total microscopic field (mm^2^) per section. H&E-stained liver sections were scored for hepatic steatosis on the level of microvesicular and macrovesicular steatosis^33^, expressed as the percentage of the total liver section area affected. Immunohistochemical detection of the macrophage marker *F4/80* was done on paraffin-embedded sections that were treated with proteinase K, using a primary Rat Anti-Mouse *F4/80* monoclonal Ab (MCA497; 1:600, Serotec, UK) and a secondary Goat Anti-Rat immunoglobulin peroxidase (MP-7444, Vector Laboratories Inc., USA). All histological and histochemical analysis were analysed using Image J software (NIH, USA).

### Liver lipids

Lipids were extracted from the liver according to a protocol from Bligh and Dyer^34^ and modified as described previously^29^. TG content was assayed as described above.

### RNA isolation and qRT-PCR

Snap-frozen liver samples were used for the extraction of RNA using a NucleoSpin RNA kit (Machery-Nagel, Germany). NanoDrop ND-1000 spectrophotometer (Isogen, The Netherlands) was used to determine concentrations and purity of RNA. Reverse transcription of RNA was done using Moloney Murine Leukemia Virus Reverse Transcriptase (Promega, The Netherlands). Gene expression levels were determined using qRT-PCR, SYBR green supermix (Biorad, The Netherlands), and gene-specific primers (Table 3). Expression of mRNA was normalized to cyclophilin (*CypA*) and ribosomal protein large P0 (*Rplp0*) RNA, and expressed as fold change versus control using the ΔΔ CT method.

**Table 3:** Primer sequences of forward and reverse primers (5′ → 3′).

Gene	Sense	Antisense
*CypA*	ACTGAATGGCTGGATGGCAA	TGTCCACAGTCGGAAATGGT
*Rplp0*	GGACCCGAGAAGACCTCCTT	GCACATCACTCAGAATTTCAATGG
*IL-10*	GACAACATACTGCTAACCGACTC	ATCACTCTTCACCTGCTCCACT
*IL-6*	AAGAAATGATGGATGCTACCAAACTG	GTACTCCAGAAGACCAGAGGAAATT
*Mcp-1*	CACTCACCTGCTGCTACTCA	GCTTGGTGACAAAAACTACAGC
*Tnf-α*	GATCGGTCCCCAAAGGGATG	CACTTGGTGGTTTGCTACGAC
*F4/80*	CTTTGGCTATGGGCTTCCAGTC	GCAAGGAGGACAGAGTTTATCGTG

### Statistical analysis

Data are presented as means ± SEM. Normal distribution of the data was tested using D’Agostino-Pearson omnibus normality test, and data were analysed with the unpaired Student’s t-test in case of normal distribution or with the nonparametric Mann–Whitney *U* test in case of not normally distributed data. Differences in body weight over time were evaluated for statistical significance by two-way ANOVA followed by Sidak’s post hoc multiple comparison test. P < 0.05 was considered as statistically significant. Analyses were performed using Graph Pad Prism version 7.0 (GraphPad Software, USA).
